# Managing prior approval for site-of-service referrals: an algorithmic approach

**DOI:** 10.1186/s12913-022-07523-3

**Published:** 2022-02-14

**Authors:** Maqbool Dada, Vishal Mundly, Chester G. Chambers, Mohammad Ali Alamdar Yazdi, Changhun Ha, Sonia E. Toporcer, Yi Zhou, Yunong Gan, Zhihua Xing, Mark Mooney, Ernest Smith, Edward Kumian, Kayode A. Williams

**Affiliations:** 1 Johns Hopkins Carey Business School, 100 International Drive, Baltimore, MD 21202 USA; 2 Department of Anesthesiology and Critical Care Medicine, Johns Hopkins School of Medicine, Baltimore, MD USA; 3 Johns Hopkins Healthcare LLC, 7231 Parkway Drivr Suite 100, Hanover, MD 21076 USA

**Keywords:** Site of service referrals, Managed care, Medicaid managed care organizations

## Abstract

**Objectives:**

Many payers and health care providers are either currently using or considering use of prior authorization schemes to redirect patient care away from hospital outpatient departments toward free-standing ambulatory surgical centers owing to the payment differential between these facilities. In this work we work with a medium size payer to develop and lay out a process for analysis of claims data that allows payers to conservatively estimate potential savings from such policies based on their specific case mix and provider network.

**Study Design:**

We analyzed payment information for a medium-sized managed care organization to identify movable cases that can reduce costs, estimate potential savings, and recommend implementation policy alternatives.

**Methods:**

We analyze payment data, including all professional and institutional fees over a 15-month period. A rules-based algorithm was developed to identify episodes of care with at least one alternate site for each episode, and potential savings from a site-of-service policy.

**Results:**

Data on 64,884 episodes of care were identified as possible instances that could be subject to the policy. Of those, 7,679 were found to be attractive candidates for movement. Total projected savings was approximately $8.2 million, or over $1,000 per case.

**Conclusions:**

Instituting a site-of-service policy can produce meaningful savings for small and medium payers. Tailoring the policy to the specific patient and provider population can increase the efficacy of such policies in comparison to policies previously established by other payers.

## Precis

We develop a rule-based algorithmic approach to enhance prior approval policies for smaller insurance providers that leverages the site of service differential to reduce costs.

## Take-away points

The site of service cost differential for outpatient services is well-documented. Managed care providers are working to develop prior authorization policies that drive care to lower cost providers. Our detailed analysis of claims data over a 15-month period for a regional provider led to the creation of an algorithm that informs decision makers on the cost implications of such policies. Findings include: 1) Average savings of $1000 per episode of care are projected for roughly 12% of such episodes; 2) Savings are maximized when the policy is provider-specific; 3) Policies for 4 CPT (Current Procedural Terminology) code families account for the bulk of the savings, and; 4) Characteristics of patient mix should inform policy construction.

## Introduction

As the cost of health care delivery continues to grow in the US, governmental and commercial payers are implementing prior authorization policies that strive to take advantage of the site-of-service (SOS) payment differential [1, 2]. Consider the cost of performing ambulatory surgical procedures. It is well documented that when performed in a hospital outpatient department (HOPD) the related payments can be substantially higher than when the same procedure is performed in a free-standing ambulatory surgical center (ASC). To contain costs, commercial payers are motivated to shift the SOS for these procedures away from HOPDs to ASCs and mobile and telehealth units. For example, in 2019 United Healthcare implemented a SOS policy that adds medical necessity criteria (MNC) to roughly a thousand surgical procedures. Under this policy, providers are required to request prior authorization to perform a procedure in the HOPD rather than at a lower cost ASC. While evidence to date suggests that there are enough safeguards in place that quality of service does not deteriorate, [3, 4] authorization to use the HOPD is automatically given for any patient with a condition of complication specified in the MNC.

Payers have a long history of using prior authorization schemes to contain drug costs [5]. However, the use of such policies to drive down payments for ambulatory surgeries is more recent. Using a national sample of claims, Higgins et al. [6]. estimated that the realized savings can range from 2.75% to as high as 25.8%. Their study also showed that SOS price differentials exist at a national level and are rising over time. Several prior works including Hayes et al. [7], and Kalidindi et al. [8]. estimate aggregate potential savings from moving some medical care from hospitals to alternate locations. However, the analysis of aggregate data does not result in the type of policy that we focus on here because such policies require analysis on a case by case basis. Detailed reports of such efforts are extremely rare.

Some national commercial payers, including United Healthcare (UHC) provide lists of procedures specified by CPT (Current Procedural Terminology) codes, tailored to zip codes, subject to its SOS cost differential policy. This suggests that smaller players could save money by mimicking such a policy. However, the fact that policies vary by zip codes suggests that results can be improved by use of policies that are matched to a region or population. Consequently, a smaller regional payer may be best served by a SOS policy tailored to their specific setting. This is natural because the payment schedule may differ, as do the health characteristics of the relevant patient populations. In addition, these smaller organizations need insights about whether the projected savings can cover the added administrative costs.

We designed this study to address how Priority Partners Managed Care Organization (PPMCO), which serves the Medicaid population in Maryland, might design its own SOS policy through analysis of its unique payment schedule and claims history. The objectives of our study included: 1) Designing a methodology to identify suitable procedures for its SOS policy; 2) Estimating the volume of cases that will be affected; 3) Providing an estimate of the potential annualized cost savings; and 4) Examining the implications of alternate design considerations that trades-off efficacy of policy implementation against potential savings.

To this end, we focus on designing a simple, rules-based algorithmic approach, to analyze the payer’s claims history. This leads to comprehensive identification of all patients that would be eligible to move. In particular, we can identify case by case, the potential alternate sites of service for each patient enabling us to provide a sharp estimate of possible savings at an episode of care level. While we focus on one payer, our algorithmic approach is universally applicable in the sense that it draws its input from claims data recorded in a standard HCFA 1500 Form.

## Methods

Our study is based on an analysis conducted for PPMCO, which is owned by Johns Hopkins HealthCare, LLC and Maryland Community Health System. This affiliation facilitated access to appropriately de-identified claims data for all 300,000-plus members served by PPMCO. In our initial analysis, we used all individual claim line items (over 10 million) from January 1, 2019, to April 23, 2020. These data provide detailed information on charges and reimbursements on behalf of all patients to all providers (facilities and professional). Since we had information in all claims, we were able to drill down precisely to identify, all eligible cases from HOPDs who could have had their procedures performed at an ASC if one offered that procedure. Conversely, we were also able to identify all procedures that could be performed by at least one ASC. By matching movable patients against eligible ASCs we were able to provide conservative estimates of potential savings.

Specifically, to develop an estimate of the potential savings involved when moving the SOS from an HOPD to an ASC, we organized the claims records into a set of episodes of care (EOCs) based on patient ID number and time of service delivery. Within each EOC, we identified the CPT (Current Procedural Terminology) code with the highest reimbursed claim entry or data element. With this in hand, we identified all ASCs that received payment for that same element. By hierarchically assigning each EOC to one CPT, we avoid duplications that could have arisen if the sorting were done at the CPT level first. This establishes a set of potentially movable cases, a set of alternate locations, and a conservative value for case-related savings in that we assumed that if an EOC were moved, only savings for that one CPT would be identified as potential savings.

Below, we specify the steps of the rules-based algorithm developed and provide an explanation of the necessity and impact of each step. We also walk through a prototypical case to highlight several nuances of our approach.

### Steps of the algorithm

- Step 1. Collect all claims data accrued for all members during the study period. Since all claims and cases are considered, there is no sampling error in the evaluation.
- Step 2. Select all instances of outpatient service that took place at an HOPD. Each line in this data set has a Place of Service code, with location “22” [9] referring to all HOPD’s. We also omit all cases handled on inpatient status.
- Step 3. Bundle claims to define EOCs. A 30-day window was used to accrue all reimbursement to a single EOC.
- Step 4. Exclude all EOCs for which total reimbursement is below $500. This helps avoid cases with minimal savings – this floor was chosen by decision makers.
- Step 5. Exclude all EOCs that are associated with an emergency room admission from 7 days before the claim to 30 days afterward [10]. Emergency room visits are unplanned, and could not have been redirected.
- Step 6. Exclude all EOCs for patients who are 18 years of age or younger. They are ineligible under outpatient general surgery precertification initiative, 2019 [11]
- Step 7. Exclude all EOCs that fall under the existing MNC. Medical Necessity Criteria are in place to direct the most difficult cases to the HOPD. Since no such policy was yet in place for this payer, the MNC policy from United Healthcare was used as a proxy.
- Step 8. Identify potential ASCs based on the most expensive procedure code. We identify ASCs based on their “Place of Service Code 24” along with the corresponding Paid Amount and CPT code.
- Step 9. Match each EOC to the candidate ASC with the largest reimbursement for the most expensive CPT code in the EOC. Matching of Outpatient EOCs with ASC records results in an expanded data set having one-to-many EOC-to-ASC combinations or “potential candidates”. Using ranking functions we select the most expensive CPT code in an EOC matched with the most expensive ASC record for a conservative savings estimate within the algorithm.
- Step 10. Exclude all EOCs with potential savings below $100. The savings needs to exceed any projected administrative cost associated with the SOS policy.

### Algorithm details

**Fig. 1:**
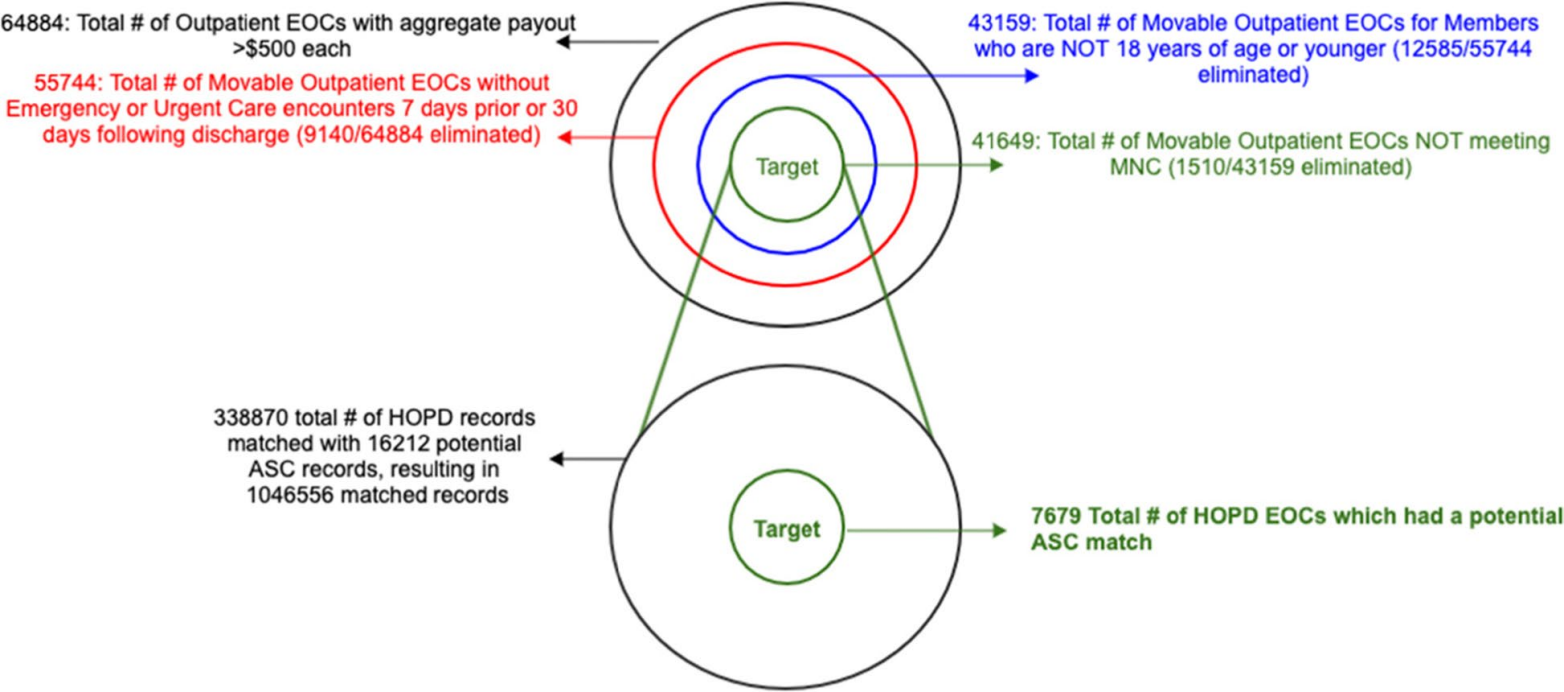
Pictorial representation of the outcomes of the algorithm. ASC, ambulatory surgical center; EOC, episodes of care; HOPD, hospital outpatient department

For our dataset, Step 1 identified roughly 10 million records for 300,000 members from January 1, 2019, to April 23, 2020. Step 2 selects a subset of 1.2 million records for outpatient services involving the HOPD. In Step 3, we bundled the claims by patient identifier and date to generate a set of EOCs. Step 4 removed EOCs with very small payments and resulted in a list of 64,884 EOCs. In Step 5, we sought to remove cases that could not be moved because they were not scheduled in advance. This step reduced the set to 55,744 EOCs. Step 6 excluded all cases for patients who are 18 years of age or younger and yielded 43,159 EOCs. Step 7 excluded all cases already covered by the MNC, and yielded 41,649 EOCs with 338,870 claim line items. Step 8 created a list of candidate ASC locations for each EOC, and identified 16,212 potential matches. In Step 9, we calculated potential cost savings for each of these EOCs. This is done using the minimum value of that savings to produce a conservative estimate. Step 10 pruned the set from Step 9 to focus on EOCs with potential savings above $100. This process yielded the final number of 7,679 EOCs. Thus from about 65,000 potential EOCs, roughly 12% (,7679) were identified as movable cases. These extractions are presented in Fig. 1 with each stage depicted by a circle of commensurate size.

Note that three aspects of this algorithm are in place to ensure that our estimate of potential savings is a conservative one. First, we only focus on the line in the claim with the highest payment. This ignores the technical fee (averaging $150) that HOPD’s add to each EOC. Second, we screen out instances where the potential savings is small, but still positive. Third, we ultimately consider the alternate location where the savings on the largest line of the claim is smallest.

**Fig. 2:**
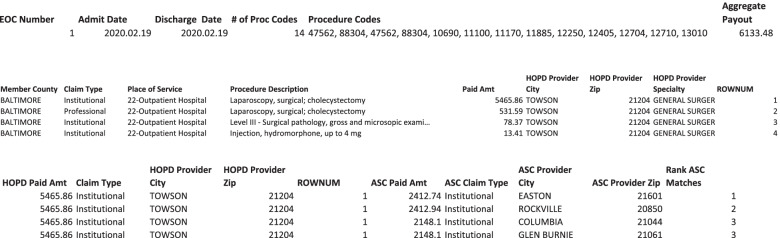
Claim Lines from Prototypical Episode of Care. EOC, episode of care; HOPD, hospital outpatient department, ROWNUM, row number

Consider the example described in Fig. 2. The figure is split into 3 parts referring to 3 extractions from the data base. The top of the figure, consistent with Step 8, shows that this EOC was defined to include 14 line items. The aggregate reimbursement for this EOC was $6133.48. The middle part of the figure, also consistent with Step 8, shows four of these items sorted in descending order based on the paid amount, and indicates that the largest payment was the institutional claim for CPT 47,562 at $5465.86. Consequently, any ASC that had billed for this CPT code was considered an alternate SOS for this EOC. Using this rule, we found 19 potential matches. The third part of the figure, consistent with Step 9, shows four of the alternate locations displayed in descending order based on the amount paid at that ASC for the same procedure. The largest payment amount among this set was $2412.74. This difference ($5,465.86—$2,114.74 = $3,351.12) is used as our estimate of potential savings for this EOC.

## Results

Execution of our 10-step procedure identified 7,679 EOCs. The total projected savings was approximately $8.2 million, or roughly $1,068 per case. The 7,679 EOCs identified involved 60 HOPDs for an average of 120 EOCs per HOPD. The savings derive from cost differentials involving 510 CPTs for an average of 15 episodes per CPT. Approximately 70% of the cases identified were not included in the UHC policy. Of the 510 unique CPT codes involved, 227 appeared on the prior approval list published by UHC, and 283 did not. The projected aggregated savings for the 227 CPTs included on the UHC list was approximately $3.1 million, or 37% of the total. Table 1 gathers results for the 10 ranges of CPT codes that appeared most often.

**Table 1:** Projected savings for cpt ranges identified most often

CPT Range Description	Count (EOC)	Payment in HOPD ($)	Fee in Alternate Location ($)	Savings ($)
Digestive system surgeries	1755	3,380,469	1,743,970	1,636,499
FGS surgeries	1420	3,057,210	1,606,792	1,450,417
MSK system surgeries	984	2,152,526	929,831	1,222,695
NULL (unidentified)	814	1,672,110	648,317	1,023,792
Integumentary surgeries	657	942,368	228,017	714,350
Office or other outpatient services	562	724,175	115,227	608,948
Eye & ocular surgeries	246	586,921	269,648	317,273
Urinary system surgeries	224	516,687	264,158	252,529
Respiratory system surgeries	168	301,607	95,896	205,711
Neurology and neuromuscular procedures	151	139,766	7,594	132,172

*EOC* Episodes of care, *FGS* Female genital system, *HOPD* Hospital outpatient department, *MSK* Musculoskeletal

Sorting the number of movable cases by CPT code revealed that 140 codes only involved a single movable case. On the other hand, 389 cases were detected for CPT 43,239 (esophagogastro-duodenoscopy). Sorting the savings by CPT code revealed an aggregate (total) savings of $12,000 for CPT 47,562 (laparoscopy), whereas roughly 700 EOCs were omitted from consideration because the each represented less than $100 of savings each. Four CPT groups—musculoskeletal, digestive, female genital, and integumentary systems, with a total of 4816 EOCs and 250 CPT codes (out of 510 unique ones)—made up over 55% of volumes and 60% of aggregate savings. Table 2 displays the procedure codes with aggregate savings of more than $50,000.

**Table 2:** Procedure codes with savings per episode of care exceeding $50,000

Procedure Code	Payment in HOPD ($)	Fee in Alternate Location ($)	EOCs per HOPD	Aggregate Savings ($)
58,558	425,786	220,978	204	204,808
20,680	306,058	126,353	90	179,705
49,650	245,954	108,930	57	137,024
29,881	174,563	81,137	66	93,426
49,505	182,003	100,772	47	81,232
45,385	191,937	112,086	137	79,851
29,888	212,960	133,367	41	79,593
57,522	141,604	70,871	86	70,733
49,585	118,102	50,764	36	67,338
43,235	116,914	51,122	114	65,792
19,120	115,428	58,517	49	56,912
29,827	139,199	84,874	37	54,325
29,880	91,161	37,178	29	53,983
67,113	104,213	51,201	30	53,012
42,420	57,863	5,684	5	52,179
52,356	148,502	96,545	52	51,957
20,610	57,065	5,600	102	51,466

*EOCs* Episodes of care, *HOPD* Hospital outpatient department

Organizing the movable EOCs by hospital revealed that 23 HOPDs accounted for 6208 of the 7679 movable cases (80%), and $6.5 million of the $8.2 million in aggregate savings (79%). At the same time, 30 HOPDs each averaged fewer than one movable case per week, and 24 averaged between one and two such cases per week. At the HOPD level, 10 CPT codes in each HOPD accounted for at least 60% of the volume and 45% of the aggregate savings at that HOPD. The list of relevant CPT codes differed by HOPD.

Not surprisingly, the potential for savings was highest among higher volume providers, which tended to be urban while the referred ASCs tended to be suburban. However, opportunities existed at smaller-volume providers as well. For example, one relatively small rural HOPD showed a potential to save $980 per movable case for a total aggregated savings of $342,665.

As Table 1 suggests, most movable cases were surgical procedures. However, several Evaluation & Mgmt. codes present significant opportunity for savings as well. For example, codes starting with 99, which are related to radiology and medical services and procedures, present significant opportunities for savings. These code groups comprise 18% of the volume and 7% of the savings, as shown in Table 3. Similar savings in office visits are also reported by Higgins et al. (2016) who used a different sampling methodology.

**Table 3:** Savings in selected procedure codes

Procedure Code	EOCs	Payment in HOPD ($)	Fee in Alternate Location ($)	Savings ($)
99,214	560	332,865	61,602	271,263
99,213	544	246,431	29,672	216,759
99,212	145	39,036	3,905	35,131
99,204	78	67,091	10,126	56,965
99,215	78	32,700	8,738	23,962
99,203	11	5,087	785	4,302
99,202	4	964	400	564
TOTAL	1420	724,175	115,227	608,948

*EOCs* Episodes of care, *HOPD* Hospital outpatient department

### Limitations

Because we used de-identified data without patient addresses, our algorithm does not account for travel distance. This work can be extended to account for travel-related costs to create an estimate of net savings; travel times and distances are indeed important but require integration with a geographic Information Systems (GIS) which would require such significant enhancements to our algorithm that it would nerit its reporting in a separate paper. The work can also be expanded to include all ASCs in the region of interest. Our work was limited to those that the payer had used over the study period. Like other studies that analyze site of service referrals, we assume that since we follow common eligibility requirements, consistent with published work discussed in the Introduction, that there is no difference in quality of care. And, 2) that demand effects would not create a surge in ASC volumes that will have an adverse impact on costs. Interestingly, the latter is unlikely in Maryland because for the Medicaid population under study, the ASC rates are set prospectively with small exceptions, while HOPDs use a fee for service approach. Thus, were costs at an ASC to increase because of SOS referrals, such an increase would result in HOPDs having to amortize costs over smaller patient volumes – this suggests that relative differences in costs would change slowly.

## Discussion and conclusions

Starting in 2019 with an initiative from UHC, several national payers began implementing prior authorization requirements for performing many procedures in HOPD’s [11]. The incentive behind these policies is that many instances exists in which the fees paid to the HOPD are greater than those paid to ASC’s for the same procedure. Unfortunately, no information is publicly available on the volume of cases and the economic savings that have been realized due to these policies. Other payers may have interest in the implementation of similar policies and should benefit from consideration of a data driven approach for their analysis, rather than relying on policies designed by national payers for their own purposes.

We used claims data from a regional managed care organization to develop a rules-based algorithm with which we could identify targets for efforts to redirect EOCs from HOPDs to alternative ASCs. By doing so, we estimated the potential volume of cases and savings that could be realized. Our analysis identified 7,679 cases and alternate SOS. Moving these EOCs would result in substantial savings. Our approach estimates this savings as $8.2 million, or over $1,000 per case. The mix of identified procedure codes varied by HOPD. As a benchmark, if the UHC prior approval policy had been used, only about 37% of this savings and 30% of cases would have been selected. This demonstrates the benefit of our algorithmic approach that yields tailored policies for implementing site of service referrals.

The algorithm presented offers a recipe for identifying CPT codes and HOPDs to prioritize in efforts to drive EOCs to lower cost locations. Our algorithm produced a conservative estimate in that it considered only the most expensive line item in the claims for an EOC, and focuses on moving the EOC to the most expensive ASC available. This evaluation can be viewed as the first step in this approach to cost reduction. When linked with ASC characteristics such as historical volume, costs and quality performance indicators, as well as travel time and distances for each patient, our approach can provide guidance on more informed decisions for patient care.

In addition, the projected savings must be weighed against a potential increase in administrative costs. For example, additional costs would arise if the payer is obligated to arrange transportation services for many of its members. In addition, there is an added burden of training providers and informing its members of the policy change. Within the context of an MCO this task may be less burdensome since primary care physicians, acting as providers, exert substantial influence in referring their patients to selected sites of service.

Such administrative costs may be best managed by focusing on the relatively small number of HOPDs and CPTs that generate the majority of savings. In theory, the payer can curate the prior authorization list specifically to the most profitable CPTs for each HOPD. An alternate approach would be to have a statewide policy that focuses on a handful of CPT groupings. Indeed, implementing a policy for the four most significant groupings would realize over 60% of the potential savings. Finally, one could consider a hybrid approach: complementing the state-wide policy with a tailored list for each of the top few HOPD’s. By limiting volume such implementations would restrain administrative costs while selecting those potential referrals with the highest potential savings.

We close by noting that our focus was on demonstrating the viability of our algorithm, it was sufficient to focus on about one year of claims history. Our approach leaves open the possibility of more in-depth analyses of dynamic patterns of referrals and cross-sectional comparisons in savings and demand across payers.

## Data Availability

The data that support the findings of this study are available from Priority Partners Medical Care Organization (PPMCO) but restrictions apply to the availability of these data, which were used under license for the current study, and so are not publicly available. Data are however available from the authors upon reasonable request and with permission of PPMCO and Johns Hopkins HealthCare.

## Abbreviations

SOS: Site of Service
HOPD: Hospital Outpatient Department
ASC: Ambulatory Surgical Centre
MNC: Medical Necessity Criteria
CPT: Current Procedural Terminology
EOC: Episode of Care
